# An Evaluation of the Occupational Health Hazards of Peptide Couplers

**DOI:** 10.1021/acs.chemrestox.2c00031

**Published:** 2022-05-09

**Authors:** Jessica C. Graham, Alejandra Trejo-Martin, Martyn L. Chilton, Jakub Kostal, Joel Bercu, Gregory L. Beutner, Uma S. Bruen, David G. Dolan, Stephen Gomez, Jedd Hillegass, John Nicolette, Matthew Schmitz

**Affiliations:** † Genentech, Inc., 1 DNA Way, South San Francisco, California 94080, United States; ‡ Gilead Sciences, Inc., Foster City, California 94404, United States; § Lhasa Limited, Granary Wharf House, 2 Canal Wharf, Leeds LS11 5PS, UK; ∥ The George Washington University, Washington, D.C. 20052, United States; ⊥ Bristol Myers Squibb, 1 Squibb Drive, New Brunswick, New Jersey 08901, United States; # Organon, Inc., 30 Hudson Street, Jersey City, New Jersey 07302, United States; ∇ Amgen Inc., One Amgen Center Drive, Thousand Oaks, California 91320-1799, United States; ○ Theravance Biopharma US, Inc., South San Francisco, California 94080, United States; ◆ AbbVie Inc., 1 North Waukegan Road, North Chicago, Illinois 60064, United States; ¶ Takeda Pharmaceutical Company Limited, 35 Landsdowne St., Cambridge, Massachusetts 02139, United States

## Abstract

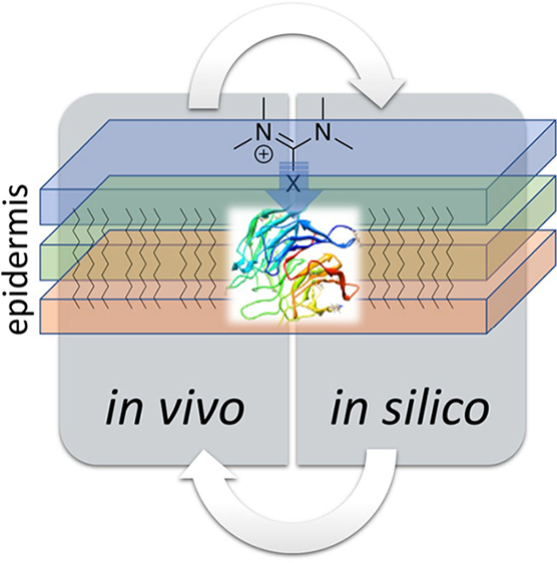

Peptide couplers (also known as amide bond-forming reagents or coupling reagents) are broadly used in organic chemical syntheses, especially in the pharmaceutical industry. Yet, occupational health hazards associated with this chemical class are largely unexplored, which is disconcerting given the intrinsic reactivity of these compounds. Several case studies involving occupational exposures reported adverse respiratory and dermal health effects, providing initial evidence of chemical sensitization. To address the paucity of toxicological data, a pharmaceutical cross-industry task force was formed to evaluate and assess the potential of these compounds to cause eye and dermal irritation as well as corrosivity and dermal sensitization. The goal of our work was to inform health and safety professionals as well as pharmaceutical and organic chemists of the occupational health hazards associated with this chemical class. To that end, 25 of the most commonly used peptide couplers and five hydrolysis products were selected for *in vivo, in vitro*, and *in silico* testing. Our findings confirmed that dermal sensitization is a concern for this chemical class with 21/25 peptide couplers testing positive for dermal sensitization and 15 of these being strong/extreme sensitizers. We also found that dermal corrosion and irritation (8/25) as well as eye irritation (9/25) were health hazards associated with peptide couplers and their hydrolysis products (4/5 were dermal irritants or corrosive and 4/5 were eye irritants). Resulting outcomes were synthesized to inform decision making in peptide coupler selection and enable data-driven hazard communication to workers. The latter includes harmonized hazard classifications, appropriate handling recommendations, and accurate safety data sheets, which support the industrial hygiene hierarchy of control strategies and risk assessment. Our study demonstrates the merits of an integrated, *in vivo* -*in silico* analysis, applied here to the skin sensitization endpoint using the Computer-Aided Discovery and REdesign (CADRE) and Derek Nexus programs. We show that experimental data can improve predictive models by filling existing data gaps while, concurrently, providing computational insights into key initiating events and elucidating the chemical structural features contributing to adverse health effects. This interactive, interdisciplinary approach is consistent with Green Chemistry principles that seek to improve the selection and design of less hazardous reagents in industrial processes and applications.

## Introduction

Allergic contact dermatitis and allergic respiratory diseases are among some of the most prevalent occupational diseases.^1^ The former accounts for an estimated 10–15% of all occupational dermal diseases, and research has shown that 9–15% of adult asthma cases are connected to occupational factors.^1,2^ Though limited information exists on their inherent hazards, case reports on occupational exposures suggest that peptide couplers (also known as amide bond-forming agents or coupling agents) are dermal and/or respiratory allergens. In fact, the first report of contact dermatitis implicated dicyclohexyl carbodiimide (DCC), a peptide coupler, in 1959.^3^ Since then, allergic contact dermatitis was observed for other peptide couplers, including diisopropyl carbodiimide (DIC), which is another common carbodiimide reagent widely used in peptide synthesis.^4−6^ Occupational allergenicity (sensitization) was reported with amidinium peptide coupling reagents, such as HATU, HBTU, HCTU, and TBTU.^7−11^ Adverse clinical signs are known to include a spectrum of respiratory symptoms, varying in severity from sneezing and runny nose to asthma and potentially life-threatening anaphylaxis. Thus, sensitized workers may no longer be able to work with or around these compounds, whether in the laboratory or on the manufacturing floor. Moreover, they can exhibit signs and symptoms when in contact with other individuals that have worked with these reagents.

**Figure 1:**
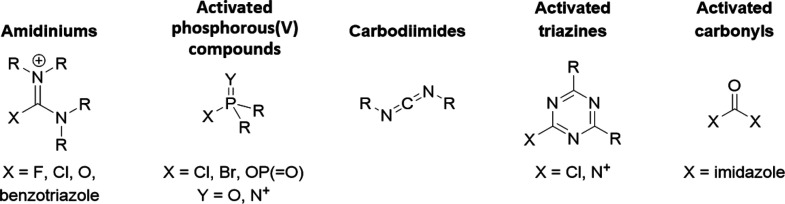
Subclasses of amide bond forming agents. The most common peptide couplers can be divided into five main subclasses, including amidiniums (amidinium salts), activated phosphorous(V) compounds (phosphonium salts), carbodiimides, activated triazines (activated heterocycles), and activated carbonyls.

Amide bonds are prevalent in organic chemical syntheses and within pharmaceutical synthesis reactions, with numerous reagents designed to facilitate amide bond formation in industrial and academic laboratories.^12−14^ The most common peptide couplers are divided into five main subclasses: amidinium salts, phosphonium salts, carbodiimides, activated triazines, and activated carbonyls (Figure 1). The ubiquity of these electrophiles and incipient nucleophiles in biological systems means that there are numerous opportunities for peptide couplers to covalently modify human proteins or other biomolecules, a phenomenon termed as haptenation. This can result in a molecular initiating event (MIE) leading to both dermal and respiratory sensitization.^15^ Given their reactive nature, peptide couplers may also cause severe dermal and eye irritation.

Prompted by reports of dermal and respiratory sensitization with peptide couplers in the literature, a pharmaceutical cross-industry task force (TF) was formed to evaluate their occupational hazards and provide guidance for this chemical class. Twenty-five peptide couplers were deemed high priority as they are widely used and handled by employees and are present in numerous pharmaceutically relevant synthetic processes (Table 1A). Occupational health hazards were assessed for each peptide coupler, including dermal irritation and corrosivity, eye irritation, and dermal sensitization. In addition, due to the known reactivity of these compounds with water, five hydrolysis products related to HBTU, TOTU, TSTU, TCFH, and TFFH were also studied to gauge whether any health hazards identified for the parent compounds may actually be ascribed to the hydrolysis product(s) (Table 1B).

**Table 1:**
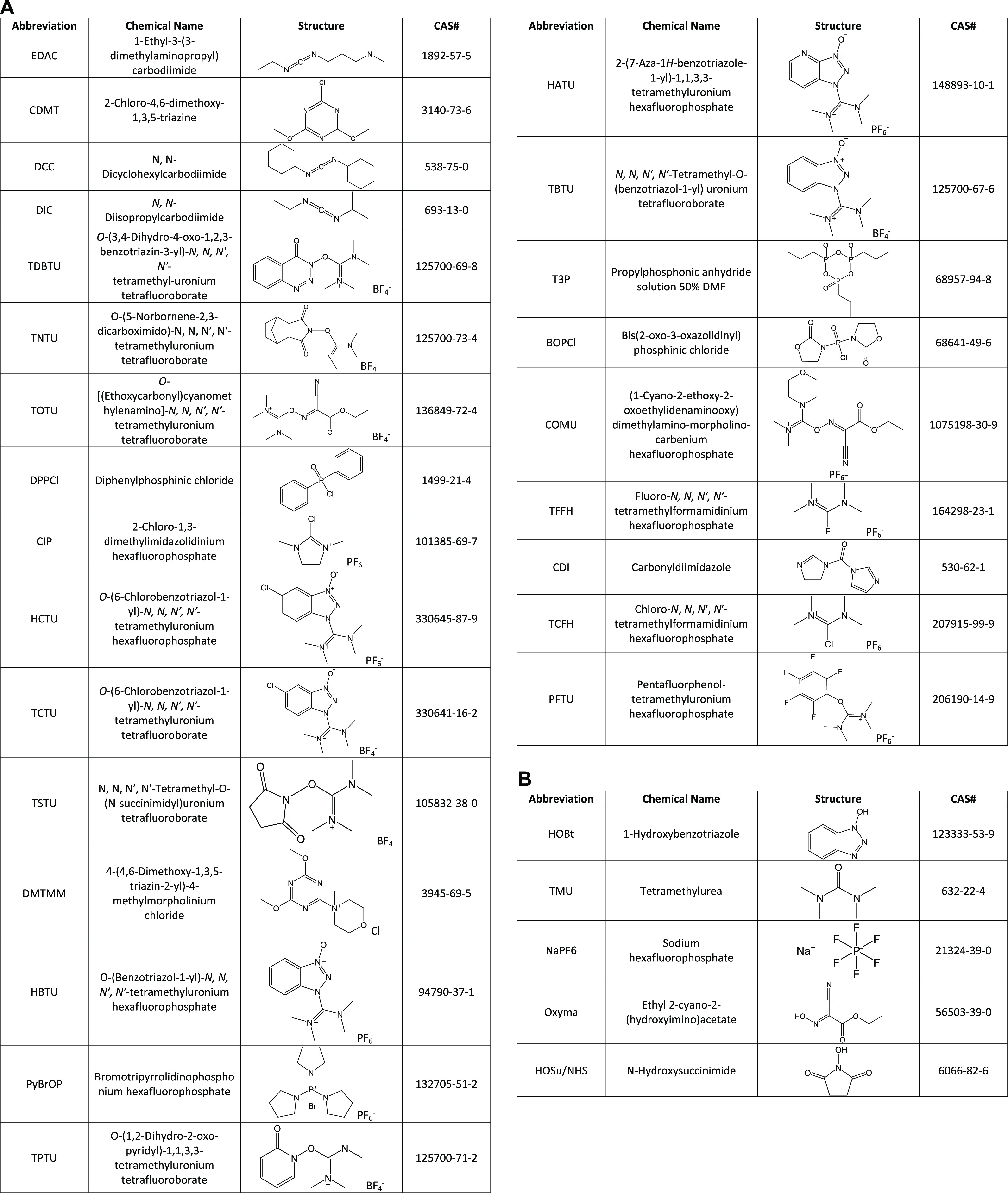
(A) Peptide Couplers Selected for Evaluation. (B) Hydrolysis Products Selected for Evaluation

As a consequence of 3R (replacement, reduction, and refinement) initiatives, there have been major advancements in *in silico*, *in vitro*, and *in vivo* models for dermal sensitization, aiding in the development of the adverse outcome pathway (AOP) for this endpoint.^16^ Dermal sensitization is an irreversible phenomenon that could result in the potential loss of employment opportunities for a sensitized chemical worker/researcher. Due to the robustness of currently available *in silico* models for dermal sensitization and because peptide couplers are understudied for their occupational health hazards, *in silico* assessments were conducted *a priori* to identify gaps in existing knowledge. Where applicable, these models were also used to gauge the sensitization potency of both peptide couplers and their hydrolysis products. This analysis prompted testing of all compounds in the *in vivo* local lymph node assay (LLNA) to assess potency of (*in silico*) predicted sensitizers and to gauge the appropriateness of existing (and the potential need to develop new) structural alerts for predicted non-sensitizers. The LLNA is a validated and fully accepted *in vivo* assay that incorporates 3R considerations such as species selection (mouse) and animal numbers (minimum number of animals to enable statistical significance). Additionally, it can be used not only for hazard identification but also prediction of potency.^17^ The latter is important for occupational safety since relative potency can aid in the selection of a peptide coupler and the determination of appropriate occupational exposure controls, including containment technology, personal protective equipment (PPE), and the development and application of safe residual surface wipe limits.^18−20^ As much as *in silico* modeling provided impetus for animal testing, LLNA results were subsequently used to inform changes in computational models. This interactive approach resulted in a horizontally integrated *in vivo*-*in silico* framework, which can be applied to reliably assess the dermal-sensitization hazard of novel peptide couplers. *In silico* models for eye and dermal irritation are currently not as well developed and therefore were not evaluated at this time. In conjunction with *in vitro* models, which were used for eye and dermal irritation/corrosivity, the present analysis offers a data-rich foundation, which is consistent with 3R considerations, and furthers our knowledge of peptide couplers as well as their selection and handling, with the potential to inform design of next-generation analogs.

## Methods

### Selection of Peptide Couplers for Testing

A TF was formed to discuss concerns around the occupational hazards presented by peptide couplers. The TF compiled a list of the most commonly used peptide couplers across participating companies (Table 1A and Table S1). These 25 compounds were deemed high priority as employees handle them often, and they are present in numerous pharmaceutical processes. Additionally, these compounds were found to lack reliable toxicological data and the hazard information on their safety data sheets (SDS) was inconsistent across suppliers (e.g., hazard classifications according to the Globally Harmonized System of Classification and Labeling [GHS]).^21^ Due to the known hydrolytic instability of peptide couplers, there is the potential that they could be hydrolyzed by ambient moisture or in the highly aqueous biological environment. Therefore, five hydrolysis products were also included to understand whether these have an influence on the health hazards attributed to and/or posed by their parent compounds (peptide couplers) (Table 1B and Tables S2–S5).

### Testing Strategy

The testing strategy utilized focused on the most common occupational illnesses reflected in the literature for peptide couplers: eye and dermal irritation/corrosivity and dermal sensitization.

### Literature Survey

A review of the literature was conducted for each of the peptide couplers and hydrolysis products prior to conducting any testing. We carried out the literature searches using the peptide coupler chemical name as well as its common abbreviated name and CAS number (Table 1A,B). Several publicly available databases were searched for testing information and occupational exposure data (Table S6). The primary goal was to find any publicly available data that would allow for appropriate classification of hazards in the handling of these compounds in an occupational or industrial setting. Briefly, the databases evaluated included the Hazardous Substances Database (HSDB), European Chemicals Agency (ECHA) database, TOXLINE, the National Toxicology Program (NTP) database, the Organisation for Economic Co-operation and Development (OECD) Screening Information Dataset (SIDS) database, and PubMed, among others. In addition to these databases, SDSs for these peptide couplers and hydrolysis products were queried to understand whether manufacturers had conducted any toxicology testing for eye and dermal irritation/corrosivity and dermal sensitization endpoints.

### *In Silico* Evaluation of Dermal Sensitization

Each compound was subjected to *in silico* analyses using deductive estimation of risk from existing knowledge (Derek) Nexus (v 6.1.0) and Computer-Aided Discovery and REdesign (CADRE; v1.4).^22−24^

Derek Nexus (Lhasa Limited, Leeds, UK, www.lhasalimited.org) is an expert knowledge-based system that uses structural alerts to provide predictions for various toxicity endpoints that are relevant to occupational health, including dermal sensitization (Derek KB 2020 1.0).^22^ A compound with a dermal sensitization alert with a likelihood of equivocal, plausible, or probable was deemed as being a positive prediction. A compound with no alerts was concluded as having a negative prediction. Negative predictions included a secondary check involving comparison of their chemical fragments against a large reference dataset of known sensitizers and non-sensitizers, to look for commonly mispredicted (misclassified) features and/or previously unseen (unclassified) features.^25^ Chemicals that were predicted to be sensitizers (positive) also had their potency predicted using a k-nearest neighbor (k-NN) model. This model identifies the most structurally and mechanistically similar nearest neighbors using an automated read-across approach to predict the chemical’s EC3 potency value (see the section on dermal sensitization studies below for a further description of the EC3 value).^26^ For chemicals that are present in the model’s training set, a “leave-one-out” approach was used to evaluate how well Derek would have predicted the EC3 value in the absence of data for the exact query chemical itself.

CADRE (DOT Consulting, LLC, www.toxfix.com) is an *in silico*, service-based platform that provides predictions for a host of mammalian and ecotoxicity endpoints. Its skin sensitization model is a tiered hybrid system that predicts dermal sensitization potential and potency by using LDA (linear discriminant analysis) models that rely on descriptors generated from mixed quantum and classical mechanics calculations and simulations of molecular interactions.^24^ In its first tier, chemicals are assessed for their ability to permeate through the stratum corneum of the dermal layer; this independent module predicts dermal permeability, *K*_p_, by considering interactions between the xenobiotic and lipid matrix components of the skin in condensed-phase Monte Carlo simulations. In the second tier, a mechanistic screen is applied to identify substructural features that correspond to known mechanisms of haptenation with dermal proteins and peptides or to moieties that can undergo metabolic activation to become potent electrophiles. In its last tier, xenobiotics are assessed for their thermodynamic and kinetic propensity to react with surface residues in dermal proteins using density functional theory (DFT) calculations. Mechanistic descriptors generated from CADRE tiers are used in statistical modeling to predict sensitization potential as well as to classify potency of the dermal sensitization response according to the ECETOC (European Centre for Ecotoxicology and Toxicology of Chemicals) system as extreme, strong, moderate, or weak (see Table S7). All model tiers consider conformational dynamics of the xenobiotic as well as its protonation states as the chemical passes from the more acidic dermal surface to the more neutral medium of the epidermis.

### Eye Irritation Studies

The bovine corneal opacity and permeability test (BCOP) was conducted on each chemical to elucidate eye irritation potential according to OECD 437.^27^ Briefly, the BCOP test method is an *in vitro* model where the test item is applied to the cornea of bovine eyes (sourced from abattoirs) and the test item’s ability to damage the corneal tissue is assessed by quantitative measurements of changes in corneal opacity and permeability. Results from this study were interpreted according to guidance in OECD 437.^27^

### Dermal Corrosion Studies

Dermal corrosion studies were conducted using either the reconstructed human epidermis (RhE) test method (according to OECD 431) or the membrane barrier test method for dermal corrosion (Corrositex; according to OECD 435).^28,29^ Two *in vitro* dermal corrosion test methods were utilized as several test items were deemed incompatible with the membrane barrier test system and were therefore carried out using the RhE test method.^28,29^

The RhE test method involves application of the test item to a three-dimensional RhE model with cultured, human-derived, epidermal keratinocytes. This model consists of organized basal, spinous and granular layers and a multi-layered stratum corneum containing intercellular lamellar lipid layers representing main lipid classes similar to those found *in vivo*. The RhE test method is based on the premise that corrosive chemicals are able to penetrate the stratum corneum by diffusion or erosion and are cytotoxic to the cells in the underlying layers. Results from this study were interpreted according to guidance in OECD 431.^28^

The *in vitro* membrane barrier test method for dermal corrosion (Corrositex) comprises two components: a synthetic macromolecular bio-barrier and a chemical detection system (CDS). This test method detects (via the CDS) membrane barrier damage caused by corrosive test chemicals following application to the surface of the synthetic macromolecular membrane barrier, presumably by the same mechanism(s) of corrosion that operate on living skin. Penetration of the membrane barrier (or breakthrough) as well as the time to breakthrough indicates potential for dermal corrosion. Results from this study were interpreted according to guidance in OECD 435.^29^

### Dermal Irritation Studies

For compounds that were negative in dermal corrosion studies, dermal irritation potential was assessed using the RhE test (see description above) according to OECD 439.^30^ The RhE model construct and premise are identical to those described previously, with the exception that the outcome of interest is dermal irritation (based on resultant cell viability) rather than corrosion. In this test method, cell viability is utilized as an indicator of dermal irritation potential. Results from this study were interpreted according to guidance in OECD 439.^30^

### Dermal Sensitization Studies

Based on the knowledge that the test items were expected to be sensitizers and the fact that their potency is of importance in understanding the occupational hazards they pose, dermal sensitization was assessed using the local lymph node assay (LLNA). At the time of writing this manuscript, *in vitro* studies are not yet able to provide a reliable potency prediction for positive compounds. LLNA experimental design, species, sex and number of animals, and procedures utilized were carried out *in vivo* according to OECD 429.^17^ Additionally, per OECD 429, a pre-screening study was included to ensure that there was no excessive irritation at the top concentration to be tested. The basic principle underlying the LLNA to determine dermal sensitization of the test material is as follows. Sensitizers induce proliferation of lymphocytes in the lymph nodes draining the site of test chemical application. This proliferation is proportional to the dose and to the potency of the applied allergen and provides a simple means of obtaining a quantitative measurement of sensitization. Proliferation is measured by comparing the mean proliferation in each test group (generally three dose groups) to the mean proliferation in the vehicle-treated control group. The ratio of the mean proliferation in each test group to that in the concurrent vehicle control group, termed the stimulation index (SI), has been judged to be indicative of a positive response when it is ≥3.^31^ The concentration corresponding to where the SI is equal to 3 is called the EC3 value (effective concentration). Thus, the lower the EC3 value, the more potent the dermal sensitizer. Results from these studies were interpreted according to guidance in OECD 429.^17^ The potencies of the dermal sensitizers were categorized based on the ECETOC system (Table S7).^32^

### Dose Selection for LLNA Studies

As potent sensitizers are of particular concern and are anticipated to pose the greatest hazard and risk of sensitization in an occupational setting, the LLNA studies were designed to detect the most potent sensitizers (strong or extreme sensitizers according to ECETOC) while minimizing animal use.^18,33^ Sensitizers with EC3 values of ≤1% are generally of greater concern from an occupational exposure and hazard perspective; thus, 1% was the top concentration studied in the majority of studies. The testing of concentrations ≤1% was intended to identify strong and extreme sensitizers; however, the top concentration in the study was based on the discretion of the sponsor (study designs for COMU, DMTMM, Oxyma, TNTU, and TSTU utilized higher maximum test concentrations). Initially, the strategy was to rely on the reduced LLNA (rLLNA) approach where a single test group is dosed at 1% and compared to a control group to see if there is a positive response at this concentration and then to proceed to conducting the full LLNA test, consisting of three concentrations at lower doses for only the positive compounds.^17^ As the majority of peptide couplers were being reported as strong sensitizers (positive at 1%) during early testing, the decision was made to change the strategy to full LLNA studies at concentrations up to and including 1%. This allowed for better potency calculations (i.e., EC3) while still reducing animal use. We should note that EC3 values were derived from the interpolation or extrapolation equations as published in Gerberick et al.^34^ Therefore, the predicted EC3 value prediction can fall outside the testing range. For example, the EC3 for a compound is extrapolated to be 1.2% as the SI is approaching 3 at the highest concentration tested of 1% (e.g., positive dose response curve with the SI = 2.8 at 1%).

### Research Ethics

All animal studies were ethically reviewed and carried out in accordance with regional directives and the associated company’s policy on the care, welfare, and treatment of animals.

## Results

### Literature Survey

While there are several case reports of sensitization reactions in humans, our survey of existing literature indicated a general lack of dermal sensitization data, such as potency information, for many of the peptide couplers and hydrolysis products evaluated. Out of the 30 compounds evaluated, only three (DCC, TFFH, and the hydrochloride [HCl] salt of EDAC [note that the freebase form of EDAC was tested as part of this project and not EDAC HCl]) were identified as dermal sensitizers in the literature; however, no dermal sensitization study or potency data were cited or located. Additionally, there was one peptide coupler (T3P) that was classified as a non-sensitizer based on the test results in the Buehler assay. Although GHS hazard classifications were identified indicating that 20 of the peptide couplers and their hydrolysis products were irritating or corrosive, there were no irritation or corrosion studies supporting these classifications for all but one compound. The only peptide coupler that was listed as irritating and corrosive in the literature based on a supportive study result (*in vivo* rabbit irritation study) was CDI. Furthermore, a review of the ECHA classification, labeling, and packaging (CLP) database revealed inconsistencies in the GHS hazard categorizations utilized for the same compound across companies. For example, the ECHA CLP database showed that >10 notifiers (manufacturers or importers) classified HBTU as an eye and skin irritant and one notifier classified it as a dermal sensitizer.^35^ These GHS classifications are included in SDSs to inform individuals handling the material(s) of the occupational health hazards they may pose.

### Dermal Irritation and Corrosion Studies

Results of the dermal irritation and corrosion studies are presented in Table 2, and their corresponding GHS classifications are presented in Table 3. Overall, 6/30 compounds tested were corrosive (4/25 peptide couplers and 2/5 hydrolysis products; GHS category 1A/B/C). Of the compounds that were not corrosive, 6/24 were dermal irritants (4/25 peptide couplers and 2/5 hydrolysis products; GHS category 2).

**Table 2:**
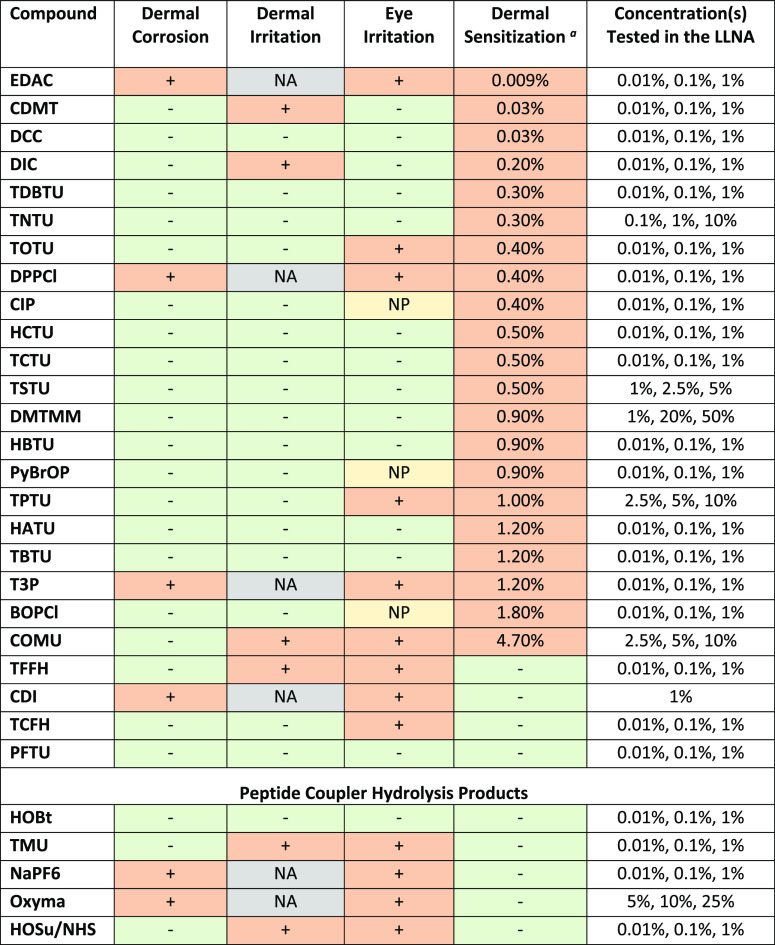
Health Hazard Study Result Summary^b^

a EC3 values are reported for compounds that were positive in the LLNA. Compounds identified as negative were concluded to be negative in the study based on the concentrations tested yet may be positive at a higher concentration.

b Symbols and acronyms: LLNA = local lymph node assay; + = positive; – = negative; NA = study not conducted since the material was determined to be corrosive; NP: no prediction can be made based on the *in vitro* study result as it was not definitively negative or positive (see OECD 437).

**Table 3:** GHS Classifications Based on Occupational Toxicology Studies^b^

Peptide Coupler	CAS No.	Dermal Sensitization GHS Category^a^	Dermal Irritation/Corrosion GHS Category	Eye Irritation GHS Category
EDAC	1892-57-5	GHS category 1A	GHS category 1A	GHS category 1
CDMT	3140-73-6	GHS category 1A	GHS category 2	NC
DCC	538-75-0	GHS category 1A	NC	NC
DIC	693-13-0	GHS category 1A	GHS category 2	NC
TDBTU	125700-69-8	GHS category 1A	NC	NC
TNTU	125700-73-4	GHS category 1A	NC	NC
TOTU	136849-72-4	GHS category 1A	NC	GHS category 1
DPPCl	1499-21-4	GHS category 1A	GHS category 1B	GHS category 1
CIP	101385-69-7	GHS category 1A	NC	NP
HCTU	330645-87-9	GHS category 1A	NC	NC
TCTU	330641-16-2	GHS category 1A	NC	NC
TSTU	105832-38-0	GHS category 1A	NC	NC
DMTMM	3945-69-5	GHS category 1A	NC	NC
HBTU	94790-37-1	GHS category 1A	NC	NC
PyBrOP	132705-51-2	GHS category 1A	NC	NP
TPTU	125700-71-2	GHS category 1A	NC	GHS category 1
HATU	148893-10-1	GHS category 1A	NC	NC
TBTU	125700-67-6	GHS category 1A	NC	NC
T3P	68957-94-8	GHS category 1A	GHS category 1C	GHS category 1
BOPCl	68641-49-6	GHS category 1A	NC	NP
COMU	1075198-30-9	GHS category 1B	GHS category 2	GHS category 1
TFFH	164298-23-1	NC (negative at ≤1%)	GHS category 2	GHS category 1
CDI	530-62-1	NC (negative at ≤1%)	GHS category 1C	GHS category 1
TCFH	207915-99-9	NC (negative at ≤1%)	NC	GHS category 1
PFTU	206190-14-9	NC (negative at ≤1%)	NC	NC
				
Peptide Coupler Hydrolysis Products				
HOBt	123333-53-9	NC (negative at ≤1%)	NC	NC
TMU	632-22-4	NC (negative at ≤1%)	GHS category 2	GHS category 1
NaPF6	21324-39-0	NC (negative at ≤1%)	GHS category 1B	GHS category 1
Oxyma	57361-81-6	NC (negative at ≤25%)	GHS category 1B	GHS category 1
HOSu/NHS	6066-82-6	NC (negative at ≤1%)	GHS category 2	GHS category 1

a For skin sensitization, potent sensitizers are identified; not classified means that the compound was concluded to be negative in the LLNA based on the concentrations tested yet may be positive at a higher concentration.

b Symbols and acronyms: NC = not classified; NP: no prediction could be made (see OECD 437).

### Eye Irritation Studies

Results of the eye irritation studies are presented in Table 2, and their corresponding GHS classifications are presented in Table 3. Overall, 13/30 compounds were eye irritants, with 9/25 of the peptide couplers and 4/5 hydrolysis products being classified as serious eye irritants (GHS category 1).

### Dermal Sensitization Studies

Results of the dermal sensitization studies are presented in Table 2, and their corresponding GHS classifications are presented in Table 3. The potency of each dermal sensitizer was categorized based on the ECETOC system (Table S7). Overall, 21/25 peptide couplers were found to be dermal sensitizers, and of these, 15 were strong or extreme (EC3 < 1%) and six were moderate sensitizers (1 ≤ EC3 < 10%). All hydrolysis products tested were non-sensitizers at concentrations at or below 1%.

### *In Silico* Results and Model Enhancements

#### Initial *In Silico* Model Performance

An overview of the initial *in silico* results for dermal sensitization is presented in Table 4. While Derek and CADRE correctly identified all or most of the compounds that were non-sensitizing based on study parameters (due to the lack of alerts), Derek missed 15 and CADRE missed six sensitizers (Table 4). When considering potency predictions, Derek and CADRE were both able to predict the correct ECETOC category for approximately one-third of the chemicals, and when they were incorrect, they were more likely to underpredict (i.e., predict the compound to be less potent than the *in vivo* data suggested) rather than overpredict.

**Table 4:**
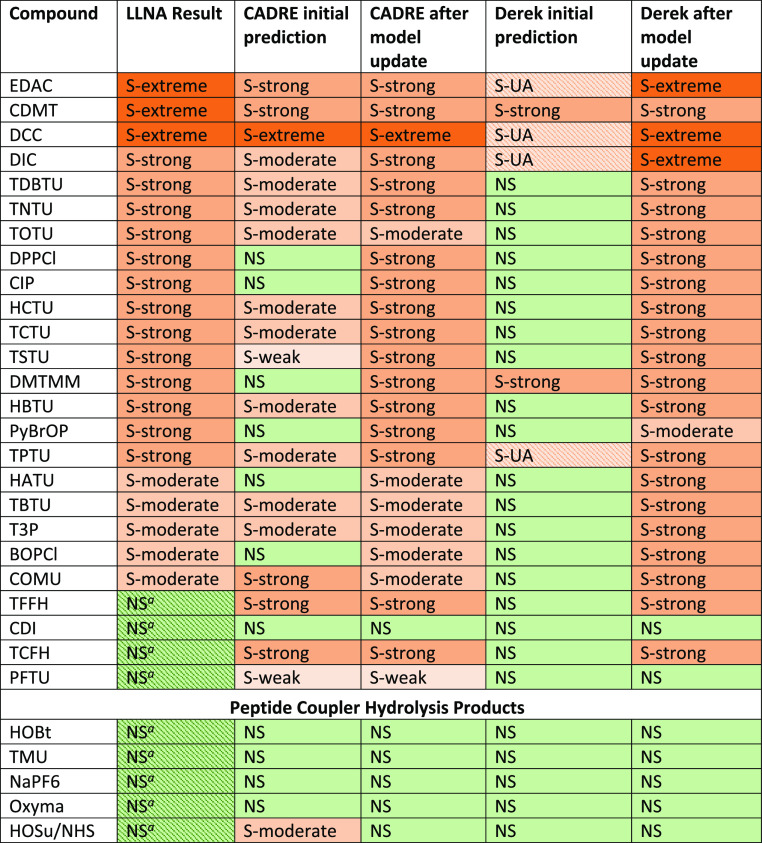
*In Silico* Model Performance for Each Compound Evaluated^b^

a Concluded to be negative in the LLNA based on the concentration(s) tested (see Table 2). The compound may be positive at higher doses.

b Abbreviations: LLNA = local lymph node assay; NS = non-sensitizer; S = sensitizer; UA = potency prediction is unavailable.

#### *In Silico* Model Updates

Upon receipt and evaluation of the dermal sensitization data from *in vivo* LLNA studies, models were revised by their respective developers, and improvements were made, including the addition of structural alerts based on LLNA results and the mechanistic understanding of haptenation for this class of chemicals.

#### Derek

Two new structural alerts were developed in Derek for the amidinium salts and activated phosphorus(V) compounds. The new amidinium alert was based on the strong sensitization results observed in three specific subclasses of these compounds: uronium salts (e.g., TDBTU, TOTU, and TNTU), guanidinium salts (e.g., TCTU, HCTU, and HBTU), and halouronium (amidinium halide) salts (e.g., CIP). These alerts were further supported by observations of occupational allergic contact dermatitis for HBTU and positive dermal prick tests for HATU, HBTU, and HCTU after a case of anaphylaxis.^9,11^ Due to the potential existence of constitutional isomers of the amidinium salts, examples being the uronium and guanidinium forms of HATU, HBTU and HCTU, both isomers were included in the alert since they are expected to be comparably electrophilic.^36,37^ The newly activated phosphorus(V) alert was based on the strong sensitization results for DPPCl and PyBrOP and the moderate sensitization results for BOPCl and T3P. The newly generated EC3 data was also incorporated into Derek’s k-NN model training set to allow potency predictions to be made within the newly defined alert spaces.

#### CADRE

In CADRE v1.5, five new structural alerts were developed to address the unique reactive moieties in the present dataset, consistent with structural clusters identified in Figure 1. While 4/5 constituted a new mechanistic moiety specific to peptide couplers, one (the activated triazine moiety) was used to augment the definition of nucleophilic aromatic substitution in the model (viz., DMTMM, which contains a quaternary amine that is a good leaving group in nucleophilic aromatic substitution but was previously not captured). As was noted for Derek, isomerism of uronium and guanidinium salts was considered in all alerts (e.g., for compounds such as HATU, the charged C=N+ fragment can be bound either to the oxygen or the ring nitrogen). Carbodiimides (alerts developed around the reactive N=C=N moiety) were incorporated both as peptide couplers and a special case of Schiff-base formers in a consensus model. It should be noted that all alerts in CADRE are mechanistic rather than structural (i.e., they are broadly defined and over-inclusive) based on the general mechanism of reactivity rather than the specific chemical structure. This is made possible by the subsequent evaluation of potency using quantum-mechanical models. Additionally, a new LDA model was developed based on the quantum-mechanical parameters of peptide coupler reactivity to improve performance in both binary- and potency-category predictions.

#### Posteriori *In Silico* Model Performance

After model updates, the resulting dermal sensitization predictions improved considerably (Table 4).

#### Derek

After the model improvements, all compounds were correctly identified as sensitizers or non-sensitizers, with the exception of TCFH and TFFH. Initially, Derek was only able to make potency predictions for 2/21 sensitizers, while after inclusion of the newly generated EC3 data, potency predictions were available for all 21/21 sensitizers. The ECETOC category was correctly predicted for 13/21 sensitizers, while six were overpredicted (i.e., predicted to be more potent than the *in vivo* data suggested), and two were underpredicted (all within one ECETOC potency category).

#### CADRE

For CADRE, with the exception of TCFH and TFFH, all compounds were correctly identified as sensitizers or non-sensitizers. PFTU was predicted to be a weak sensitizer and may be a sensitizer in the LLNA if tested at higher concentrations. In terms of potency, 18/21 sensitizers were correctly predicted by the ECETOC category; none were overpredicted, and three were underpredicted post-improvements (all within one ECETOC category of potency).

## Discussion

Although there are several case studies of allergic contact dermatitis attributed to peptide couplers, we found little information available (e.g., *in vitro* or *in vivo* study results) on their occupational health hazards. There were also inconsistencies in health hazard categorizations such as GHS categorizations presented in SDSs and the ECHA CLP database. These inconsistencies and the lack of data supporting or refuting many of these hazards can result in a risk to employees handling these chemicals. Given the severity of the sensitization reactions reported in the literature, the lack of sensitization potency data and data on other common occupational hazards (e.g., eye and dermal irritation and corrosion), we carried out a series of toxicological studies to fill in these data gaps for each of the commonly used peptide couplers and hydrolysis products (Table 1A,B). Crucially, the lack of available data also impeded the ability of *in silico* dermal sensitization models to make accurate binary and potency predictions (Table 4). The present analysis improves the status quo by facilitating advancements in these two computational models for the endpoint of dermal sensitization and provides information for training other predictive tools for this endpoint and this chemical class.

Given the inherent reactivity of peptide couplers, dermal sensitization and eye and dermal irritation were expected to be potential health hazards. We found that dermal corrosion and irritation (40%; 12/30 compounds) as well as eye irritation (43%; 13/30) were health hazards associated with nearly half of the peptide couplers tested as well as their hydrolysis products (Tables 2 and 3). Dermal sensitization study results (determined via the LLNA) established that the primary hazard of concern for peptide couplers is their sensitization potential, as shown in Table 2. Most of the peptide couplers tested (21/25) were sensitizers, of which 15 were strong or extreme (EC3 < 1%) and six were moderate sensitizers (EC3: 1.0–4.7%). As the focus of this effort was to identify peptide couplers that are strong and extreme sensitizers, higher concentrations (e.g., >1%) were generally not tested. Therefore, for those that were considered non-sensitizers (4/25 peptide couplers), there is a possibility that they could be positive if tested at higher concentrations, resulting in a weak or moderate response. This information is key when comparing outcomes of the LLNA with *in silico* predictions in Table 4.

Due to their potential for rapid hydrolysis, the hydrolysis products of several peptide couplers were tested to understand whether the hazards observed (e.g., sensitization) were due to the hydrolysis product(s) rather than the peptide coupler itself. Our results confirm that hydrolysis products owing to their reduced electrophilic reactivity are not central to the mechanism of sensitization as none of them were positive at or below a concentration of 1%, in contrast to their parent compounds (Table 2 and Tables S2–S5). To that end, the likely mechanism of dermal sensitization for peptide couplers is intrinsically linked to the compounds’ innate electrophilicity as well as their ability to transform carboxylic acids into reactive electrophiles. Our data highlights challenges around the safe use of peptide couplers and the development of safer analogs as their intrinsic reactivity is required for applications in organic synthesis but is likely to lead to undesirable occupational hazards, such as dermal sensitization. We envision that the health hazard data generated in this study can be used in conjunction with process safety information on peptide couplers to provide a more holistic understanding of the requirements necessary to protect workers using these chemicals.^38^ This information can also be utilized in alignment with Green Chemistry principles that seek to develop less hazardous chemical syntheses (Principle 3) by improving the selection of less hazardous reagents in chemical processes and applications.^49^ Given the widespread usage of peptide couplers in the pharmaceutical industry as well as academia, elucidation of their occupational health hazards is critical to ensuring that workers are aware of the hazards and can mitigate them (e.g., through the use of exposure controls and PPE). Identifying the proper GHS hazard categorizations for each of these compounds with regard to dermal sensitization, dermal corrosion/irritation, and eye irritation (Table 3) is a step in the right direction to enabling the accurate and more harmonized communication of the occupational health hazards posed.

### *In Silico* Model Improvements

As occupational health hazard data was lacking for peptide couplers, *in silico* models were also expected to have room for improvement when predicting hazards for this chemical class. Several commercial and publicly available *in silico* models are available with varying levels of predictive accuracy.^23,24,39,40^ We therefore sought to utilize the information garnered to improve *in silico* models utilized for initial hazard predictions, specifically with regard to sensitization predictivity (including potency estimations).

Five distinct structural clusters were observed within the set of peptide couplers tested, which are outlined in Figure 1. These moieties consisted of amidiniums (halouroniums, uroniums, and guanidiniums) (*n* = 15), activated phosphorus(V) compounds (*n* = 4), carbodiimides (*n* = 3), activated triazines (*n* = 2), and activated carbonyls (*n* = 1). Inspection of the *in vivo* sensitization data within these clusters revealed that each cluster had broadly similar sensitization potencies (Figure S1), thus adding credence that the proposed clusters are likely to have similar toxicity mechanisms. A few exceptions to these trends were observed in the amidinium subclass of reagents. The amidinium halides TFFH and TCFH were non-sensitizing at a dose of 1% in the LLNA (although a slight dose–response was observed) as was the uronium PFTU, though no dose–response was observed for this chemical. The remaining five compounds were various hydrolysis products from the reaction of well-known peptide couplers, which were all non-sensitizing at ≤1%.

The initial performance of *in silico* models was largely hindered by the lack of underlying data and supportive structural alerts for peptide couplers. Additionally, the high intrinsic reactivity of peptide couplers was found to be out of domain in existing LDA models within CADRE, which resulted in potency underpredictions (Table 4). The latter point underscores one of the key challenges in developing a robust predictive model—the need for a balanced training set, which is often lacking for highly reactive chemical classes.^41^ Thus, our initial assessment showed that there was room for improvement in *in silico* models for this class of compounds. To that end, efforts were undertaken to improve the models to recognize key features of peptide couplers responsible for the sensitization reactions, leveraging existing mechanistic knowledge about the reactivity of these chemicals.

Subsequent to model improvements, a boost in performance was observed in both tools. From Table 4, concordance between *in silico* models and the LLNA increased owing to the incorporation of newly generated *in vivo* data (in the form of additional model alerts: two in Derek and five in CADRE) and retraining of the statistical models (LDA in CADRE and k-NN in Derek). Due to limitations of testing at 1% in the LLNA, to identify moderate/weak sensitizers, Table 4 represents a horizontally integrated *in silico*-*in vivo* analysis, where consensus is deemed more important than perceived hierarchy in driving hazard-based decisions. For example, based on *in silico* evaluations, it is suspected that TFFH and TCFH are in fact true sensitizers (predicted to be strong sensitizers in both models). While both TFFH and TCFH were identified as negative at 1% in the LLNA, their intrinsic reactivity suggests that they are likely potent sensitizers and may be identified as such at higher test concentrations in the LLNA. This is further supported by read-across from the strong sensitizer, CIP (EC3 = 0.4%), which is highly structurally similar to TFFH and TCFH and contains the same reactive moiety.

### Incorporating New Mechanistic Knowledge in *In Silico* Models

It is important to recognize that any *in silico* model can be improved to fit (new) experimental data, but whether these changes increase the robustness of the model is of far greater concern. The consensus among computational toxicologists is that robustness, i.e., model dependability beyond training-set data, is largely driven by the model’s mechanistic underpinning.^41,42^ In that regard, both Derek and CADRE check the proverbial box based on their existing architectures; in contrast to statistically heavy models, they are rooted in the underlying chemistry that drives molecular interactions in toxicological pathways. The structural alerts used by both models capture the mechanistic requirements for biotransformations that lead to the skin sensitization response. To that end, we expect that the newly developed alerts for peptide couplers (described in more detail below) will offer robust predictivity for future compounds in this class particularly when supplemented by quantum-mechanical modeling (CADRE) or k-nearest neighbor modeling (Derek) to gauge structural nuances between analogs. In addition, negative predictions from Derek are supported by a structural fragmentation approach that highlights features associated with a lower performance and/or increased uncertainty to help users assess the reliability of any predictions of inactivity.^25^ In CADRE, confidence scores, derived from computational approximations and parametrical similarity to training-set compounds, are provided alongside predictions as a gauge of uncertainty in both positive and negative outcomes. The discussion below briefly outlines how changes were made to these programs to promote credibility in their robustness.

It is important to note that, for peptide couplers, their mechanism of function overlaps with that of toxicity in the sensitization AOP. These chemicals are designed to be very reactive, and so, their potency appears relatively insensitive to substitution.^43^ However, basic physical-organic principles still apply, and substitutions that increase the electrophilicity of the reactive center, decrease steric bulk, and/or increase the acidity of the leaving group can increase toxicity. In many cases, these trends can be elucidated by relating parameters determined during *in silico* testing with the *in vivo* sensitization data obtained from LLNA studies. For example, HCTU/TCTU are more potent than HATU/TBTU due to the electron-withdrawing chlorine substituent on the aromatic ring, which makes the former more electrophilic and thus more reactive. In CADRE, this is effectively captured by the electrophilicity index, which is greater for HCTU/TCTU (5.95 eV) than for HATU/TBTU (5.77 eV). Thus, while the alert itself does not make the potency distinction in CADRE, the quantum-mechanical model, which relies on a host of electronic and steric parameters descriptive of the entire toxicant as well as its moieties and atoms, does.

Reactivity is not the sole driver of sensitization potency as skin permeability also plays a role. CADRE integrates a skin permeability coefficient (*K*_p_) prediction via its CADRE-KP module, which is based on mixed quantum and molecular mechanics simulations of the chemical’s behavior in various compartments of the stratum corneum. We observed that the predicted average log *K*_p_ is higher for extreme sensitizers (−4.9) than strong sensitizers (−5.5) and moderate sensitizers (−7.4) across the peptide-coupler dataset, which is consistent with previous reports.^24^

In Derek, the two new alerts developed were based on the two clusters containing sensitizers that were not already covered by the model. One new alert was developed for amidinium reagents based on strong sensitization results for the specific subclasses of uronium (e.g., TDBTU, TOTU, and TNTU), guanidinium (e.g., TCTU, HCTU, and HBTU), and amidinium halide salts (e.g., CIP). The mechanism of sensitization for this subclass is likely the nucleophilic attack of amine residues within skin proteins to the highly electrophilic carbon of the C–N double bond.^44^ Additionally, an alert was developed for activated phosphorus(V) compounds, where the mechanism of sensitization is expected to be nucleophilic substitution by carboxylate groups in peptide side chains at the phosphorous with release of a suitable leaving group to yield a mixed phosphorous-carbon anhydride, which can proceed to react further with other nucleophiles.^45,46^

The hydrolytic instability of these peptide couplers, which can affect the amount of active compound that reaches its biological target, is another aspect of their reactivity, which is arguably harder to assess. When these compounds enter a highly aqueous biological environment, a competition between a reaction with a biological target, leading to sensitization, and a hydrolysis reaction, leading to a relatively inert non-sensitizing compound, occurs. This can be illustrated by comparing coupling reagents that are effective peptide couplers in aqueous environments (EDAC and TSTU) with compounds that are rapidly hydrolyzed and only used under strictly anhydrous conditions (TCFH and TFFH). EDAC and TSTU are strong sensitizers compared to TCFH and TFFH, which are not sensitizing at 1% in the LLNA. Focusing on hydrolysis may also help explain why TFCH and TFFH are strongly sensitizing in the *in silico* models but not strongly sensitizing in the *in vivo* studies, where hydrolysis may mask these hazards. We should note that computational models have the capacity to distinguish between electrophilic reactivity of peptide couplers with water and surface residues in skin proteins, which are generally considered to be softer nucleophiles.

### Incorporating Occupational Health Hazard Data into the Peptide Coupler Selection Process

Although most of the peptide couplers tested were sensitizers, the results differentiated those that are extreme and strong sensitizers from moderate sensitizers, thus allowing a potential selection of the least hazardous peptide coupler where possible. Additionally, the potency of a sensitizer can be used to guide handling practices via hazard communications to industrial safety professionals as well as workers who are manufacturing or handling them. In designing a synthetic transformation using these peptide couplers, factors such as yield, product purity, and reaction rate are typically the key drivers for selecting which compound will be used. However, with the data presented here, the sensitization potency can now be considered as an additional selection criterion that will lessen occupational hazards when comparing two peptide couplers that may have similar performance by all the standard metrics used by chemists. To that end, peptide couplers are listed in order of decreasing sensitization potency in Table 2. The sensitization hazard may drive handling requirements as selection of a peptide coupler with higher sensitization potency may require additional protection from chemical exposure to reduce occupational risk. For this reason, the sensitization hazard could be a key factor in the design of safer synthetic processes in the future.

### A Collaboration that Benefits Companies and Employees

While *in silico* model development is traditionally unidirectional, i.e., experimental data informs model development, there are clear benefits to a more collaborative process. In such a scenario, experimentalists are prompted to carry out new experiments to fill data gaps identified by modelers as crucial to the stability and robustness of their predictive tools. This establishes a *de facto* “two-way street” between the experiment and *in silico* model, which iteratively improves the latter as well as points at deficiencies and uncertainties of the former.^42^ Previous discussion about the reactivity and potency of TFFH and TCFH highlights how *in silico* models can inform experiments; *in vivo* testing of amidinium and carbodiimide reagents underscores the complexity of this analysis and shows how critical data gaps in computational models can be filled with experimental data. Bringing together subject matter experts across toxicology study and *in silico* model design, implementation and result interpretation are critical to the advancement and expansion of understanding to enable informed future decisions.

A certain level of openness and trust in data-sharing is key to sustain such a collaborative effort: Companies handling animal data on proprietary compounds would benefit from divulging sensitive information to modelers, and modelers could gain from discussing the limitations of their models. Commercial competitiveness is critical for both parties involved in the process, but it should not come at the cost of hindering progress and advancing our collective knowledge. As this study demonstrates, a collaborative effort can effectively improve *in silico* models, avoid duplication of effort, and thus reduce costs, resource strain, and the use of animals. Overall, this advances the effort to limiting and, perhaps one day, eliminating most animal testing and is aligned with the mission of the 3Rs (reduction, refinement, and replacement of animal studies).^47,48^

## Conclusions

Peptide couplers are reagents used in amide bond formation, which is of particular interest to the pharmaceutical industry; however, their occupational hazards have not yet been systematically characterized. Here, we evaluated the occupational health hazards of 25 representative peptide couplers and a select group of their hydrolysis products to fill this knowledge gap using *in vivo, in vitro*, and *in silico* models. Our findings confirm that dermal sensitization is of concern for this chemical class, as is the potential for eye and dermal irritation and corrosivity. Our work highlights the overall benefit that results from a concerted effort across functions, involving toxicologists, computational modelers, and chemists. We showed that, together, we can more effectively elucidate health hazards, improve *in silico* models, and inform safer choices in chemical development and the chemical research space across all stakeholders in industry and academia. Most importantly, a cross-disciplinary collaboration that rests on transparency in data sharing and data generation is necessary to achieve system-based hazard evaluations that are consistent with 3Rs and Green Chemistry principles for the evaluation, selection and design of safer chemicals and products.

## Supporting Information Available

Figure S1. Structural clusters observed within the set of peptide couplers, together with their general sensitization potencies in the LLNA; Table S1. Information on compounds tested; Table S2. HBTU with hydrolysis products; Table S3. TOTU with hydrolysis products; Table S4. TSTU with hydrolysis products; Table S5. TCFH/TFFH with hydrolysis products; Table S6. Literature search strategy; and Table S7. ECETOC categories for skin sensitization (PDF)

## Abbreviations

3Rs: reduction, refinement, and replacement of animal studies
AOP: adverse outcome pathway
BCOP: bovine corneal opacity and permeability
BOPCl: bis(2-oxo-3-oxazolidinyl)phosphinic chloride
PyBrOP: bromotripyrrolidinophosphonium hexafluorophosphate
CADRE: computer-aided discovery and redesign
CDI: carbonyldiimidazole
CDMT: 2-chloro-4,6-dimethoxy-1,3,5-triazine
CDS: chemical detection system
CIP: 2-chloro-1,3-dimethylimidazolidinium hexafluorophosphate
CLP: classification, labeling, and packaging
COMU: (1-cyano-2-ethoxy-2-oxoethylidenaminooxy)dimethylamino-morpholino-carbenium hexafluorophosphate
DCC: *N*,*N*-dicyclohexylcarbodiimide
Derek: deductive estimation of risk from existing knowledge
DFT: density functional theory
DIC: *N,N*-diisopropylcarbodiimide
DMTMM: 4-(4,6-dimethoxy-1,3,5-triazin-2-yl)-4-methylmorpholinium chloride
DPPCl: diphenylphosphinic chloride
EC3: effective concentration where the simulation index is equal to 3
ECETOC: European Centre for Ecotoxicology and Toxicology of Chemicals
ECHA: European Chemicals Agency
EDAC: 1-ethyl-3-(3-dimethylaminopropyl)carbodiimide
GHS: Globally Harmonized System of Classification and Labelling
HATU: 2-(7-aza-1*H*-benzotriazole-1-yl)-1,1,3,3-tetramethyluronium hexafluorophosphate
HBTU: *O*-(benzotriazol-1-yl)-*N,N,N*′*,N*′-tetramethyluronium hexafluorophosphate
HCTU: *O*-(6-chlorobenzotriazol-1-yl)-*N,N,N*′*,N*′-tetramethyluronium hexafluorophosphate
HSDB: Hazardous Substances Database
k-NN: k-nearest neighbor
*K*_p_: permeability coefficient
LDA: linear discriminant analysis
LLNA: local lymph node assay
MIE: molecular initiating event
NTP: National Toxicology Program
OECD: Organisation for Economic Co-operation and Development
PFTU: pentafluorphenol-tetramethyluronium hexafluorophosphate
PPE: personal protective equipment
rLLNA: reduced local lymph node assay
RhE: reconstructed human epidermis
SDS: safety data sheets
SI: stimulation index
SIDS: screening information dataset
T3P: propylphosphonic anhydride solution 50% DMF
TBTU: *N,N,N*′*,N*′-tetramethyl-*O*-(benzotriazol-1-yl)uronium tetrafluoroborate
TCFH: chloro-*N*,*N*,*N*′,*N*′-tetramethylformamidinium hexafluorophosphate
TCTU: *O*-(6-chlorobenzotriazol-1-yl)-*N,N,N*′*,N*′-tetramethyluronium tetrafluoroborate
TDBTU: *O*-(3,4-cihydro-4-oxo-1,2,3-benzotriazin-3-yl)-*N,N,N*′*,N*′-tetramethyl-uronium tetrafluoroborate
TF: task force
TFFH: fluoro-*N,N,N*′*,N*′-tetramethylformamidinium hexafluorophosphate
TPTU: *O*-(1,2-dihydro-2-oxo-pyridyl)-1,1,3,3-tetramethyluronium tetrafluoroborate
TNTU: *O*-(5-norbornene-2,3-dicarboximido)-*N*,*N*,*N*′,*N*′-tetramethyluronium tetrafluoroborate
TOTU: *O*-[(ethoxycarbonyl)cyanomethylenamino]-*N,N,N*′*,N*′-tetramethyluronium tetrafluoroborate
TSTU: *N*,*N*,*N*′,*N*′-tetramethyl-*O*-(*N*-succinimidyl)uronium tetrafluoroborate
