# Supplementary material for: An Evaluation of the Occupational Health Hazards of Peptide Couplers
Source: Chem Res Toxicol. 2022 May 9;35(6):1011–22. doi: 10.1021/acs.chemrestox.2c00031 (PMC9214767; doi:10.1021/acs.chemrestox.2c00031)
Supplement: Supplementary file 1 — tx2c00031_si_001.pdf [file tx2c00031_si_001.pdf]

Supporting Information for:

An Evaluation of the Occupational Health Hazards of Peptide Couplers

Jessica C. Graham[*,†,1], Alejandra Trejo-Martin[†,2], Martyn L. Chilton[3], Jakub Kostal[4], Joel Bercu[2], Gregory L. Beutner[5], Uma S. Bruen[6], David G. Dolan[7], Stephen Gomez[8], Jedd Hillegass[5], John Nicolette[9], Matthew Schmitz[10]

1. Genentech, Inc., 1 DNA Way, South San Francisco, CA 94080
2. Gilead Sciences, Inc., Foster City, CA 94404
3. Lhasa Limited, Granary Wharf House, 2 Canal Wharf, Leeds, LS11 5PS, UK
4. The George Washington University, NW Washington, District of Columbia 20052
5. Bristol Myers Squibb, 1 Squibb Drive, New Brunswick, NJ 08901
6. Organon, Inc., 30 Hudson Street, Jersey City, NJ 07302
7. Amgen Inc., One Amgen Center Drive, Thousand Oaks, CA 91320-1799
8. Theravance Biopharma US, Inc., South San Francisco, CA 94080
9. AbbVie Inc., 1 North Waukegan Road, North Chicago, IL 60064
10. Takeda Pharmaceutical Company Limited, 35 Landsdowne St, Cambridge, MA 02139

[†] Co-first authors

* Corresponding author

Table of Contents:

Figure S1. Structural clusters observed within the set of peptide couplers, together with their general sensitization potencies in the LLNA -- pg. 2
Table S1. Information on Compounds Tested --- pg. 3
Table S2. HBTU with Hydrolysis Products --- pg. 4
Table S3. TOTU with Hydrolysis Products --- pg. 4
Table S4. TSTU with Hydrolysis Products --- pg. 5
Table S5. TCFH/TFFH with Hydrolysis Products -- pg. 5
Table S6. Literature Search Strategy -- pg. 6
Table S7. ECETOC Categories for Skin Sensitization -- pg. 6

Figure S1. Structural clusters observed within the set of peptide couplers, together with their general sensitization potencies in the LLNA.

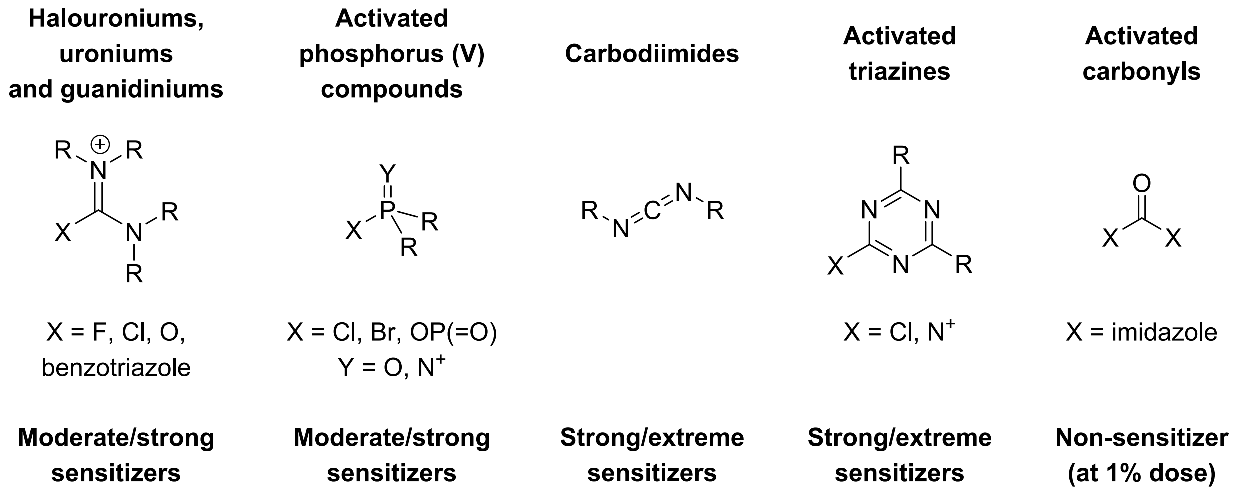

Table S1. Information on Compounds Tested

Abbreviation	CAS No.	Supplier	Product / Catalog No.	Purity (%)
BOPCl	68641-49-6	Sigma-Aldrich	15140	≥97
CDI	530-62-1	Sigma-Aldrich	115533	Reagent grade
CDMT	3140-73-6	Oakwood Products, Inc.	067544	99.3
CIP	101385-69-7	Sigma-Aldrich	420336	98
COMU	1075198-30-9	Sigma-Aldrich	712191	97
DIC	693-13-0	Sigma-Aldrich	38370	98
DCC	538-75-0	Sigma-Aldrich	D80002	>95
DMTMM	3945-69-5	Sigma-Aldrich	74104	96
DPPCl	1499-21-4	Sigma-Aldrich	230235	98
EDAC	1892-57-5	Sigma-Aldrich	39391	≥97
HATU	148893-10-1	Sigma-Aldrich	445460	97
HBTU	94790-37-1	Sigma-Aldrich	12804	98
HCTU	330645-87-9	Alfa Aesar	H26402	98
HOBt	123333-53-9	Sigma-Aldrich	711489	97
NaPF6	21324-39-0	Sigma-Aldrich	208051	98
NHS (HOSU)	6066-82-6	Sigma-Aldrich	130672	98
Oxyma	57361-81-6	Sigma-Aldrich	37347	≥99
PFTU	206190-14-9	Combi-Blocks, Inc.	QA-0814	99
PyBrOP	132705-51-2	Sigma-Aldrich	18565	95
T3P	68957-94-8	Sigma-Aldrich	81801	50.2
TBTU	125700-67-6	Sigma-Aldrich	12806	97
TCFH	207915-99-9	Combi-Blocks, Inc.	QA-9362	98
TCTU	330641-16-2	Tokyo Chemical Industry (TCI) CO.,LTD.	C1926	99.9
TDBTU	125700-69-8	Tokyo Chemical Industry (TCI) CO., LTD.	D3263	99.8
TFFH	164298-23-1	Combi-Blocks, Inc.	QA-7984	98
TMU	632-22-4	Sigma-Aldrich	T24503	99
TNTU	125700-73-4	AAPPTEC	CXZ043	99.3
TOTU	136849-72-4	Merck KGaA	8.51088.0025	≥99
TPTU	125700-71-2	Sigma-Aldrich	37347	99
TSTU	105832-38-0	Sigma-Aldrich	385530	97

Table S2. HBTU with Hydrolysis Products

Abbreviation	Structure	CAS#	LLNA EC3 %
HBTU (parent compound)	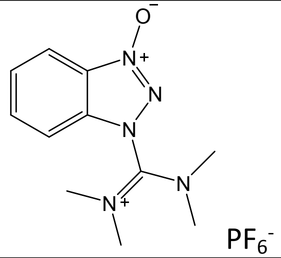	94790-37-1	0.9
HOBt	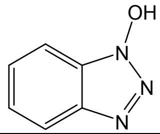	123333-53-9	Negative at 1%
TMU	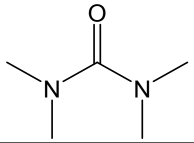	632-22-4	Negative at 1%
NaPF6	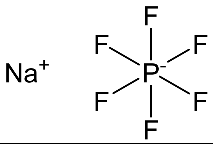	21324-39-0	Negative at 1%

Table S3. TOTU with Hydrolysis Products

Abbreviation	Structure	CAS#	LLNA EC3 %
TOTU (parent)	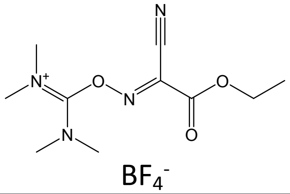	136849-72-4	0.4%
Oxyma	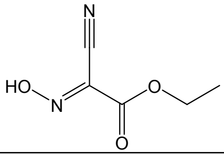	57361-81-6	Negative at 25%
TMU	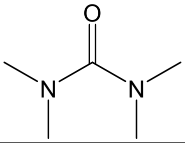	632-22-4	Negative at 1%

Table S4. TSTU with Hydrolysis Products

Abbreviation	Structure	CAS#	LLNA EC3 %
TSTU (parent)	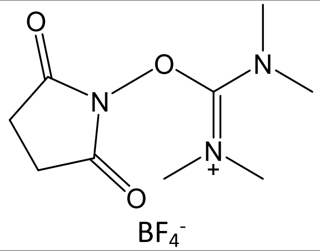	105832-38-0	0.5%
HOSu/NHS	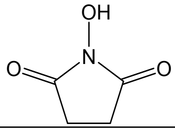	6066-82-6	Negative at 1%
TMU	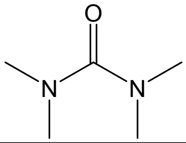	632-22-4	Negative at 1%

Table S5. TCFH/TFFH with Hydrolysis Products.

Abbreviation	Structure	CAS#	LLNA EC3 %
TFFH (parent)	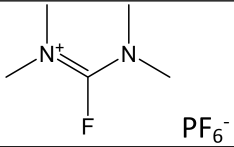	164298-23-1	Negative at 1%
TCFH (parent)	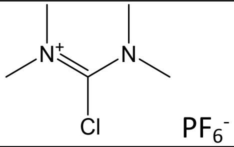	207915-99-9	Negative at 1%
TMU	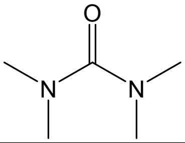	632-22-4	Negative at 1%
NaPF6	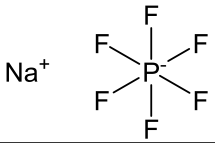	21324-39-0	Negative at 1%

Table S6. Literature Search Strategy

**Databases searched using: CAS number, compound name and compound abbreviated name**	
HSDB:	https://pubchem.ncbi.nlm.nih.gov/
TOXLINE:	https://www.ncbi.nlm.nih.gov/pubmed?term=tox%20%5Bsubset%5D%20AND%20
NTP:	https://ntp.niehs.nih.gov/publications/reports/index.html
Toxplanet:	https://www.toxplanet.com/index.html
ECHA/REACH:	http://echa.europa.eu/information-on-chemicals/registered-substances<br>http://echa.europa.eu/web/guest/information-on-chemicals/cl-inventory-database
SIDS:	http://webnet.oecd.org/HPV/UI/Search.aspx
HERO database:	http://hero.epa.gov
PubMed:	http://www.ncbi.nlm.nih.gov/pubmed

Table S7. ECETOC Categories for Skin Sensitization

ECETOC Category	EC3 Value (%)
Weak	≥10%
Moderate	≥1 - <10%
Strong	≥0.1 - <1%
Extreme	<0.1%
